# Immediate implant-based breast reconstruction following mastectomy is not associated with delays in adjuvant therapy

**DOI:** 10.3389/fonc.2025.1715130

**Published:** 2026-01-02

**Authors:** Lilian Paz, Jorge Biazús

**Affiliations:** 1 Postgraduate Program in Health Sciences: Gynecology and Obstetrics, Federal University of Rio Grande do Sul, Porto Alegre, Brazil; 2 Department of Breast Surgery, Aristides Maltez Hospital, Salvador, Brazil

**Keywords:** breast cancer, mastectomy, breast reconstruction, implant-based breast reconstruction, adjuvant chemotherapy, adjuvant radiotherapy, delay treatment, time to treatment

## Abstract

**Background:**

Immediate breast reconstruction (IBR) following mastectomy is an integral component of breast cancer surgery and contributes to improved quality of life. However, its association with higher postoperative complication rates has raised concerns about potential delays in the initiation of adjuvant therapy, which may negatively impact oncologic outcomes. This study aimed to evaluate whether immediate implant-based breast reconstruction affects the timing of adjuvant treatment.

**Methods:**

This retrospective cohort included 930 women with stage I–III invasive breast cancer who underwent mastectomy between 2018 and 2022. Patients were stratified into two groups based on surgical approach: mastectomy with IBR (n = 200) and mastectomy without IBR (n = 730). The primary outcome was time from definitive cancer surgery to initiation of the first adjuvant treatment (chemotherapy or radiotherapy). A delay was defined as an interval exceeding 12 weeks. Logistic regression models were used to assess factors associated with treatment delay.

**Results:**

The cohort was composed predominantly of Black women (91%). Women who underwent IBR initiated adjuvant therapy earlier than those without reconstruction [11.0 (IQR 8.0–14.0) vs. 12.0 (IQR 9.0–16.0) weeks; p < 0.01], with fewer experiencing delays beyond 12 weeks (33.3% vs. 47.4%, p < 0.01). While IBR was associated with lower odds of delay in univariate analysis (OR 0.55; 95% CI, 0.39–0.76), this association was attenuated and not statistically significant after adjustment in the multivariable analysis (OR 0.69; 95% CI, 0.46–1.01). Independent predictors of delay included clinical stage III and surgery during the COVID-19 pandemic (2020–2022). Delays were more pronounced among patients scheduled for radiotherapy.

**Conclusion:**

In this real-world cohort with predominant representation of Black women, immediate implant-based breast reconstruction after mastectomy did not delay the initiation of adjuvant therapy. IBR can be safely integrated into breast cancer treatment planning without compromising timely access to care, although factors such as advanced disease stage and healthcare workflow remain key contributors to treatment delays. Registry: Brazilian Clinical Trials Registry (ReBEC), RBR-3h369zf.

## 1 Introduction

Breast cancer remains the most common malignant neoplasm among women worldwide, with an estimated 2.3 million new cases reported in 2022 (1). In Brazil, projections for 2025 anticipate 73,610 new cases, underscoring the disease’s substantial burden at the national level (2). Among those diagnosed, approximately 40% to 45% require mastectomy, particularly in cases of locally advanced disease. In early clinical stages (I and II), mastectomy is indicated in roughly one-third of patients; in more advanced stages (III and IV), this proportion rises to nearly two-thirds (3–5).

Immediate breast reconstruction has become an integral part of surgical planning and is endorsed by the European Society for Medical Oncology (ESMO), except in specific contraindications such as inflammatory breast cancer (6). Immediate breast reconstruction offers a range of benefits, including a single surgical procedure, reduced hospitalization, lower overall healthcare costs, preservation of body image, and improvements in self-esteem and psychological well-being—all of which contribute to better quality of life for patients (7–10).

Among the various reconstructive approaches, implant-based breast reconstruction is frequently employed and suitable for a broad patient population. It is generally faster, associated with shorter postoperative recovery times, and presents lower morbidity compared to autologous techniques (9). However, implant-based IBR has also been linked to increased postoperative complications, such as infections and reoperations, when compared to mastectomy alone (11–13).

These potential complications have raised concern among clinicians regarding whether IBR might contribute to delays in initiating adjuvant therapy—delays that could, in turn, compromise oncologic outcomes. Although there is no universal consensus on the optimal timeframe for starting adjuvant treatment, evidence suggests that delays beyond recommended windows may negatively impact survival (14). Despite these concerns, current evidence on whether implant-based IBR following mastectomy actually delays the initiation of adjuvant therapy remains inconclusive and often conflicting. This gap in the literature underscores the need for further investigation.

In this context, the present study aimed to evaluate whether implant-based IBR following mastectomy delays the time to adjuvant therapy—chemotherapy or radiotherapy—compared to mastectomy without reconstruction.

## 2 Materials and methods

### 2.1 Study design and setting

This retrospective cohort study was conducted involving women diagnosed with invasive breast carcinoma in clinical stages I to III, according to the 8th edition of the American Joint Committee on Cancer (AJCC) (15). These patients underwent mastectomy at Aristides Maltez Hospital, a philanthropic tertiary oncology referral center located in Salvador, Brazil, which exclusively serves patients through the Public Health System, between January 2018 and December 2022. The study was approved by the Research Ethics Committee of the Institute of Health Sciences at the Federal University of Bahia (approval number 5.584.821) and registered with the Brazilian Clinical Trials Registry (ReBEC) under number RBR-3rxj6y6.

### 2.2 Participants and data collection

A total of 1,839 medical records were reviewed. Inclusion criteria consisted of women aged 18 years or older who underwent mastectomy followed by adjuvant chemotherapy or radiotherapy. Exclusion criteria included metastatic disease, prophylactic mastectomy, bilateral surgery, tumor recurrence, and initiation of adjuvant therapy at another institution. Patients who underwent pedicled myocutaneous flap reconstruction were also excluded to maintain a homogeneous study population. Additionally, cases in which adjuvant therapy was initiated more than 180 days after surgery were excluded, as they do not reflect standard clinical practice. Data were manually extracted from electronic medical records. A total of 930 women met the eligibility criteria and were divided into two groups: those who underwent IBR with implants (n = 200, 21.5%) and those who had mastectomy alone (n = 730, 78.5%). The study design followed the STROBE (Strengthening the Reporting of Observational Studies in Epidemiology) guidelines (16).

### 2.3 Outcomes

The primary outcome variable, time to adjuvant therapy, was defined as the time interval between the last definitive cancer surgery and the initiation of the first adjuvant treatment, either chemotherapy or radiotherapy. The final cancer surgery could include additional oncologic procedures, such as margin re-excision or axillary lymph node dissection, but excluded reoperations due to surgical complications. The initiation of adjuvant therapy was determined by the date of the first cycle of adjuvant chemotherapy or the first session of radiotherapy, whichever occurred first. For patients who received both modalities, only the start date of the first administered treatment was considered. Time to hormone therapy was not assessed. A delay in adjuvant therapy was defined as an interval exceeding 12 weeks after surgery. Although the European Society for Medical Oncology (ESMO) (6) recommends initiating adjuvant systemic therapy ideally within 4–6 weeks after surgery, this broader threshold was adopted based on evidence showing that delays beyond 8–12 weeks are associated with worse oncologic outcomes. Previous studies have reported that initiating adjuvant chemotherapy after this period increases the risk of recurrence and mortality, with hazard ratios ranging from 1.5 to 1.6 for overall survival and significantly higher recurrence rates (14, 17, 18). The same criterion was used to define delay in the initiation of postoperative radiotherapy, as recommended by the 1st Central-Eastern European Professional Consensus Statement on Breast Cancer (19). The time to initiation of endocrine therapy was not included in the analysis, as its administration is often less standardized and typically occurs sequentially after chemotherapy or radiotherapy, precluding a consistent postoperative interval definition. Subgroup analyses were conducted based on the type of first adjuvant therapy. Time to chemotherapy (from surgery to first cycle) and time to radiotherapy (from surgery to first session) were analyzed as secondary outcomes, given their distinct clinical implications. Therefore, these endpoints were examined in separate analytical subsections.

### 2.4 Variables

Sociodemographic variables included age at surgery, ethnicity, menopausal status, smoking status, presence of comorbidities (hypertension, diabetes mellitus, cardiovascular, pulmonary, neurological, and psychiatric conditions), body mass index (BMI), year of surgery, and distance (in kilometers) from the patient’s city of residence to the hospital city, calculated using Google Maps. Clinical and pathological variables included clinical stage, Tumor histologic type, histologic grade, hormone receptor status, HER2 expression, Ki-67 proliferation index, skin invasion, lymphovascular invasion, *in situ* component and extranodal extension. Surgical variables included type of mastectomy—total mastectomy (complete breast removal), skin-sparing mastectomy (removal of breast tissue and nipple-areola complex while preserving most of the skin), and nipple-sparing mastectomy (removal of breast tissue while preserving the skin and nipple-areola complex); axillary surgery (sentinel lymph node biopsy or axillary lymph node dissection) and implant type (direct-to-implant or tissue expander). Oncological treatment variables included neoadjuvant chemotherapy, adjuvant therapy, adjuvant chemotherapy regimen, and radiotherapy modality (conventional or hypofractionated).

### 2.5 Statistical analysis

Statistical analyses were conducted using IBM SPSS Statistics, version 23 (IBM Corp., 2023). Continuous variables were expressed as means (± standard deviations) or medians (interquartile ranges). Categorical variables were reported as absolute frequencies and percentages. Between-group comparisons were conducted using Student’s *t*-test or the Mann–Whitney *U* test for continuous variables, depending on distribution, and the chi-square test for categorical variables. The time from surgery to initiation of adjuvant therapy (in weeks) was treated as a continuous variable. Univariate analysis included sociodemographic, clinical, pathological, and surgical variables, selected based on clinical relevance and evidence from literature. Variables with a *p*-value < 0.10 in univariate analysis were entered for inclusion in the multivariate model. A two-tailed *p*-value < 0.05 was considered statistically significant.

### 2.6 Sample size calculation

The sample size was estimated using the Sealed Envelope tool (Sealed Envelope Ltd., 2021), based on a retrospective cohort study by Grigor et al. (20), which found no significant difference in time to initiation of adjuvant therapy between patients who underwent mastectomy alone and those who received IBR following mastectomy. Assuming a 95% confidence level, a 5% significance level, and an expected difference of 5%, a minimum of 90 participants (45 in each group) would be required. Nevertheless, all eligible patients treated between 2018 and 2022 were consecutively included through a nonprobability convenience sampling approach, ensuring the real-world representativeness of the sample.

## 3 Results

### 3.1 Study population characteristics

**Figure 1:**
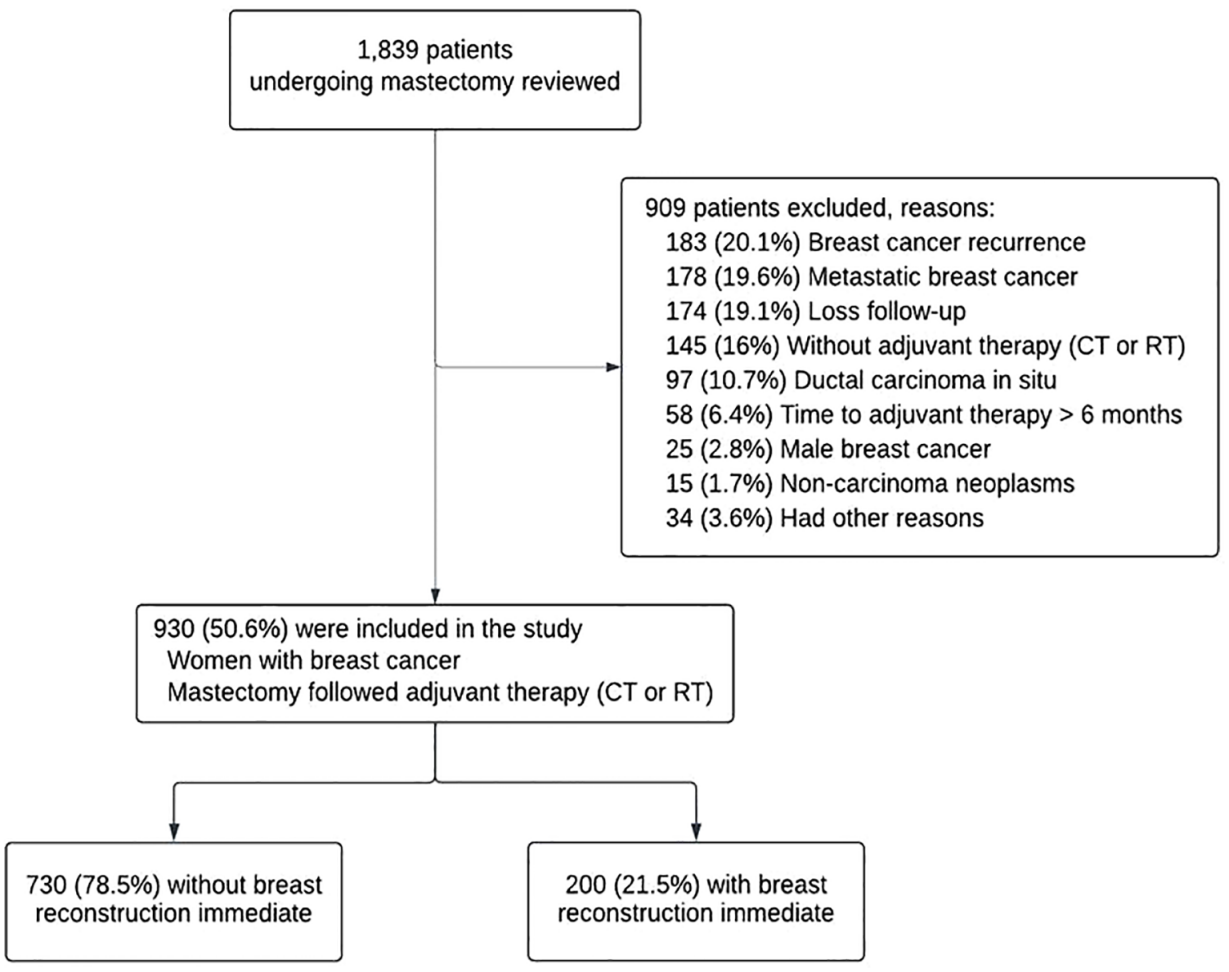
Flowchart of patient selection and exclusion. CT, chemotherapy; RT, radiotherapy.

Of the 1,839 patients initially assessed, 930 women with stage I–III breast cancer who underwent mastectomy followed by chemotherapy or radiotherapy at Aristides Maltez Hospital were included in the study. A total of 909 patients (49.4%) were excluded, primarily due to cancer recurrence, metastatic disease, loss to follow-up, or absence of adjuvant chemotherapy or radiotherapy (**Figure 1**).

The study population consisted predominantly of Black women (91%), with a median age of 50 years (IQR 42–60). Most were premenopausal. A greater proportion of patients presented with advanced T3/T4 tumors (445/831; 53.6%), and the most common immunohistochemical subtype was HR+/HER2− (540/916; 59%). Neoadjuvant chemotherapy was administered to 478 patients (51.4%). Baseline characteristics are summarized (**Table 1**).

**Table 1:** Baseline characteristics of the study population stratified by immediate breast reconstruction.

Characteristics	All patients n = 930	No IBR^a^ n = 730	IBR n = 200	*P*-value
	No (%)	No (%)	No (%)	
Age, years				< 0.01
Median (IQR^b^)	50 (42 - 60)	53 (44 - 63)	44 (37- 49)	
Ethnicity				0.36
Black	846 (91.0)	667 (91.4)	179 (89.5)	
White	55 (5.5)	36 (4.9)	15 (7.5)	
Missing	33 (3.5)	27 (3.7)	6 (3.0)	
Distance from city of residence to hospital city, km, (n = 916)				0.85
< 23	392 (42.8)	302 (42.1)	90 (45.2)	
≧ 23 to < 56	66 (7.2)	52 (7.3)	14 (7.0)	
≧ 56 to < 361	231 (25.2)	185 (25.8)	46 (23.1)	
≧ 361	227 (24.8)	178 (24.8)	49 (24.6)	
Menopausal status, (n = 850)				< 0.01
Premenopause	435 (51.2)	292 (43.6)	143 (79.0)	
Postmenopause	415 (48.8)	377 (56.4)	38 (21.0)	
Smoking status, (n = 910)				0.01
Never	737 (81.0)	564 (79.1)	173 (87.8)	
Current	75 (8.2)	68 (9.5)	7 (3.6)	
Former	98 (10.8)	81 (11.4)	17 (8.6)	
Comorbidities				< 0.01
No	531 (57.1)	381 (52.2)	150 (75.0)	
Yes	399 (42.9)	349 (47.8)	50 (25.0)	
BMI^c^, kg/m², (n = 668)				**< 0.01**
Median (IQR)	26.8 (23.7 - 30.3)	27.1 (23.8 - 30.5)	25.3 (23.1 - 28.1)	
BMI classification				0.01
Underweight	10 (1.5)	8 (1.6)	2 (1.4)	
Normal weight	227 (34.7)	162 (31.8)	65 (44.8)	
Overweight	240 (36.6)	188 (36.9)	55 (35.9)	
Obesity	178 (27.2)	152 (29.8)	26 (17.9)	
Clinical stage, (n = 834)				< 0.01
I	63 (7.6)	36 (5.4)	27 (16.1)	
II	395 (47.4)	295 (44.3)	100 (59.5)	
III	376 (45.1)	335 (50.3)	41 (24.4)	
Neoadjuvant chemotherapy				0.02
No	478 (51.4)	360 (49.3)	118 (59.0)	
Yes	452 (48.6)	370 (50.7)	82 (41.0)	
Year of surgery				< 0.01
2018	220 (23.7)	181 (24.8)	39 (19.5)	
2019	153 (16.5)	113 (15.5)	40 (20.0)	
2020	185 (19.9)	142 (19.5)	43 (21.5)	
2021	198 (21.3)	140 (19.2)	58 (29.0)	
2022	174 (18.7)	154 (21.1)	20 (10.0)	
Axillary surgery				< 0.01
SLNB^d^	238 (25.6)	152 (20.8)	86 (43.0)	
ALND^e^	692 (74.4)	578 (79.2)	114 (57.0)	

a IBR, immediate breast reconstruction;

b IQR, interquartile range;

c BMI, body mass index;

d SLNB, sentinel lymph node biopsy;

e ALND, axillary lymph node dissection; Statistically significant differences (p < 0.05) are shown in bold.

A total of 200 women (21.5%) underwent IBR. This group was significantly younger, with a median age of 44 years (IQR 37–49), had a higher prevalence of premenopausal status (79.0% vs. 43.6%; *p* < 0.01), and fewer comorbidities (25.0% vs. 47.8%; p < 0.01). Subpectoral reconstruction was performed in all patients in this group. The implant types used were direct silicone implants in 134 patients (67.0%), tissue expanders in 53 (26.5%), and unspecified in 13 (6.5%). Mastectomy types in this group included skin-sparing in 107 patients (53.5%), nipple-sparing in 28 (14.0%), and total mastectomy in 65 (32.5%).

Of the 730 women (78.5%) who underwent mastectomy without IBR, there was a higher prevalence of obesity (29.8% vs. 17.9%; *p* = 0.01), clinical stage III disease (50.3% vs. 24.4%; *p* < 0.01), axillary lymph node dissection (79.2% vs. 57%; *p* < 0.01), and receipt of neoadjuvant chemotherapy (50.7% vs. 41.0%; *p* = 0.02). Subsequent postoperative histopathological findings, including tumor type and biological markers, are detailed (**Table 2**).

**Table 2:** Postoperative histopathological characteristics.

Characteristics	All patients n = 930	No IBR^a^ n = 730	IBR n = 200	*P*-value
	No (%)	No (%)	No (%)	
Histologic type				< 0.01
IDC^b^	851 (91.5)	675 (92.5)	176 (88.0)	
ILC^c^	46 (4.9)	27 (3.7)	19 (9.5)	
Others	33 (3.5)	28 (3.8)	5 (2.5)	
Histologic grade, (n = 879)				< 0.01
1	85 (9.7)	57 (8.2)	28 (15.0)	
2	480 (54.6)	375 (54.2)	105 (56.1)	
3	314 (35.7)	260 (37.6)	54 (28.9)	
Immunohistochemical subtype, (n = 916)				0.05
HR^d^+ HER^e^2-	540 (59.0)	412 (57.3)	128 (65)	
HR+ HER2+	97 (10.6)	72 (10.0)	25 (12.7)	
HR- HER2-	183 (20.0)	154 (21.4)	29 (14.7)	
HR- HER2+	96 (10.5)	81 (11.3)	15 (7.6)	
Ki-67 proliferation index, (n = 841)				0.29
< 14	247 (29.4)	184 (28.1)	63 (33.9)	
≧ 14 to < 20	70 (8.3)	57 (8.7)	13 (7.0)	
≥ 20	524 (62.3)	414 (63.2)	110 (59.1)	
*In situ* component				0.11
Absent	523 (56.2)	421 (57.7)	102 (51.0)	
Present	407 (43.8)	309 (42.3)	98 (49.0)	
Lymphovascular invasion				0.80
Absent	764 (82.2)	598 (81.9)	166 (83.0)	
Present	166 (17.8)	132 (18.1)	34 (17.0)	
Skin invasion				0.03
Absent	856 (92.0)	664 (91.0)	192 (96.0)	
Present	74 (8.0)	66 (9.0)	8 (4.0)	
Extranodal extension				0.01
Absent	866 (93.1)	671 (91.9)	195 (97.5)	
Present	64 (6.9)	59 (8.1)	5 (2.5)	

a IBR, immediate breast reconstruction;

b IDC, invasive ductal carcinoma;

c ILC, invasive lobular carcinoma;

d HR, hormone receptor;

e HER2, human epidermal growth factor receptor 2. Statistical significance was defined as p < 0.05 and is shown in bold.

Among patients receiving chemotherapy as their initial adjuvant treatment, 424 (45.6%) received this modality, with anthracycline-based regimens administered in 319 (75.2%) cases and taxanes in 296 (69.8%). Of the remaining 506 (54.4%) who initiated adjuvant therapy with radiotherapy, a hypofractionated protocol was used in 165 (32.6%) cases. Chemotherapy was more commonly used as the initial adjuvant therapy in the IBR group, occurring in 110 (55.0%) women, whereas radiotherapy predominated among those without IBR, with 416 (57.0%) receiving it as their initial modality.

### 3.2 Time to adjuvant therapy

The mean time from surgery to initiation of the first adjuvant therapy was 12.3 ± 4.6 weeks. A total of 412 patients (44.3%) experienced delays greater than 12 weeks, more commonly in the non-reconstruction group (47.4% vs. 33.3%; *p* < 0.01). In the overall population, patients in the IBR group initiated adjuvant therapy earlier than those in the non-IBR group [11.0 (IQR 8.0–14.0) vs. 12.0 (IQR 9.0–16.0) weeks; p < 0.01].

**Figure 2:**
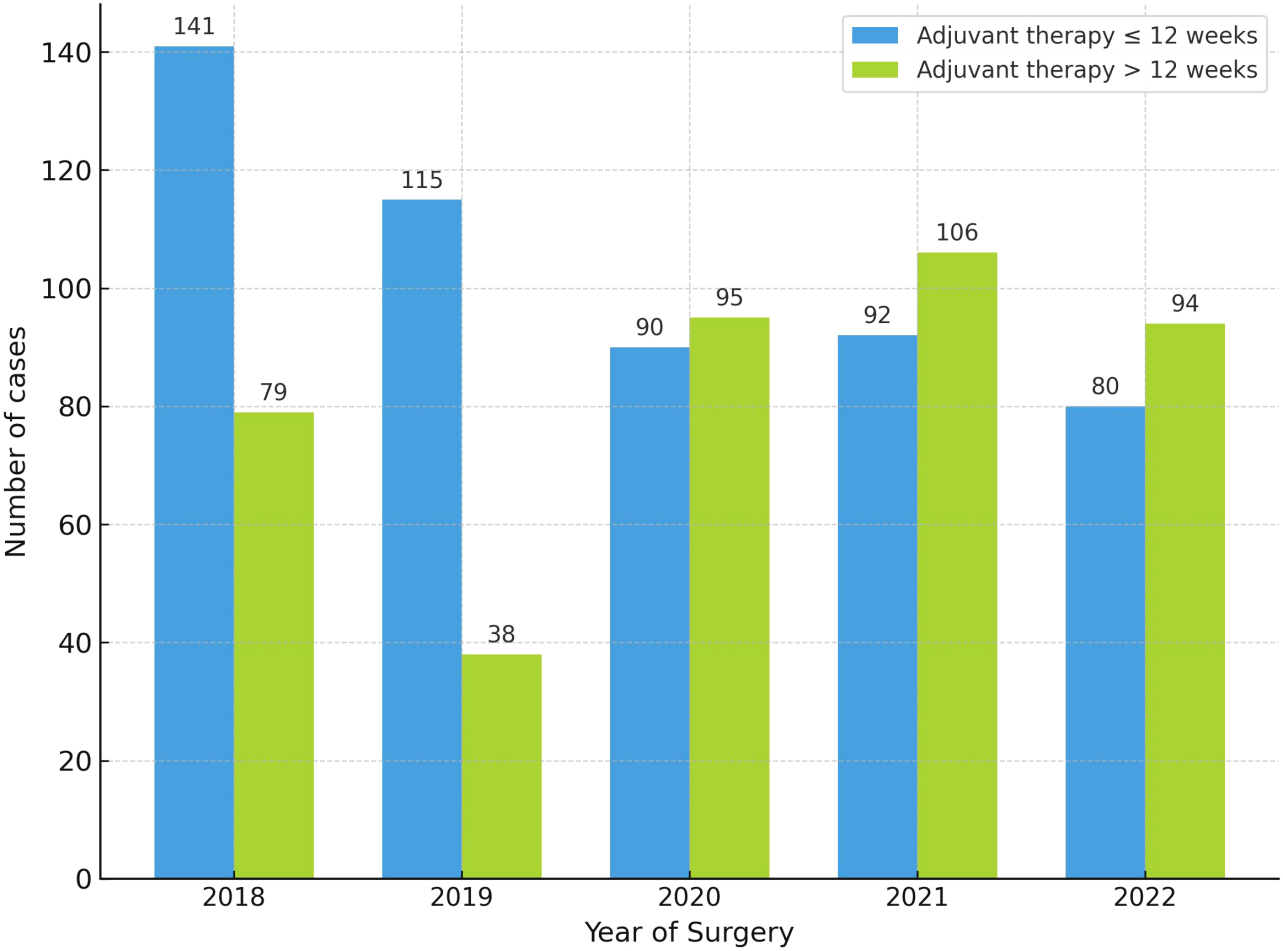
Distribution of patients (n = 930) by year of surgery and timing of adjuvant therapy initiation (≤12 vs. >12 weeks). Bars represent the number of patients who started adjuvant therapy within ≤12 weeks (blue) or after >12 weeks (green) of surgery for each year. Numbers above the bars indicate the number of cases in each category.

**Figure 3:**
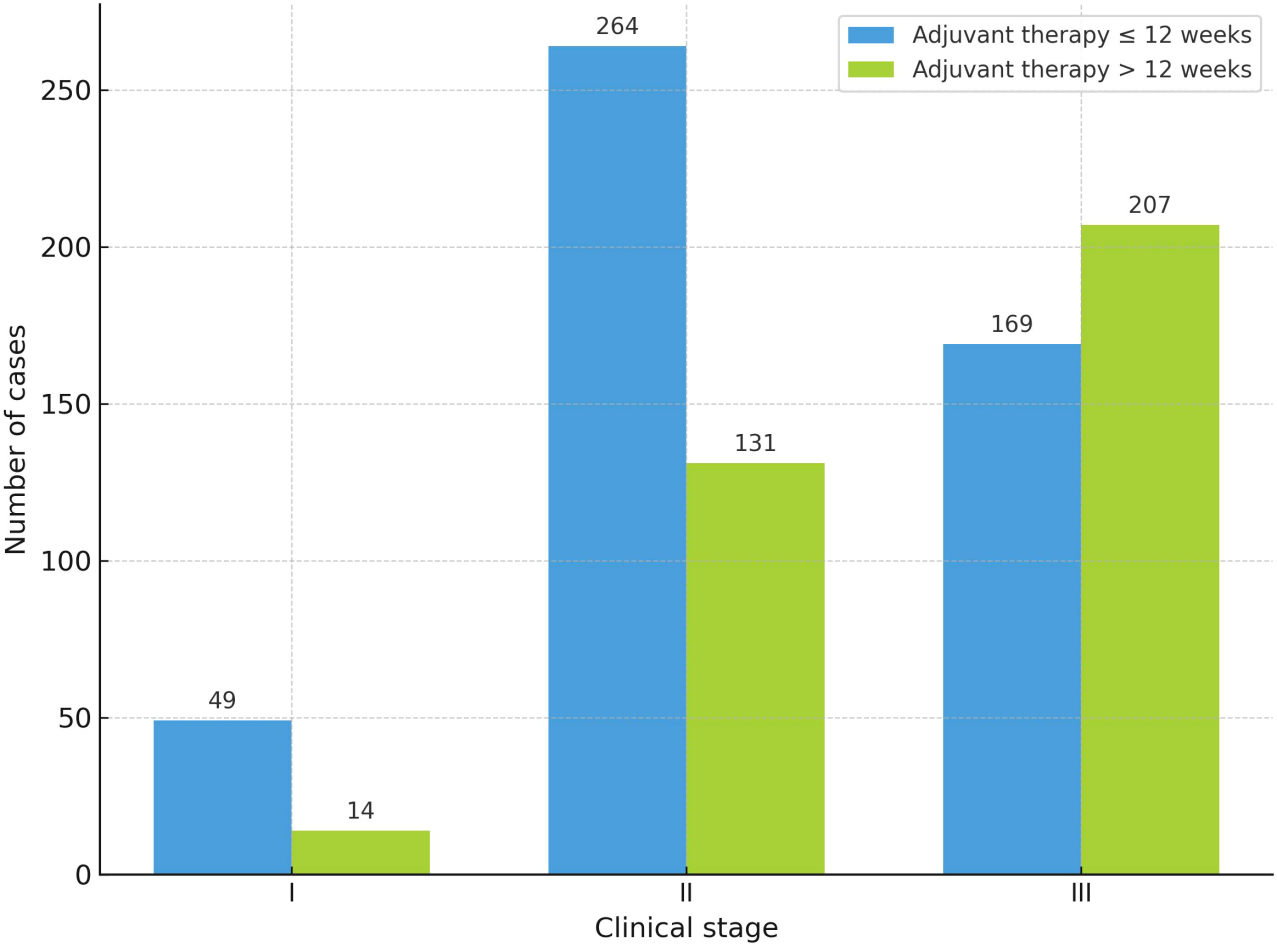
Distribution of patients (n = 834) by clinical stage and timing of adjuvant therapy initiation. Each pair of bars represents the number of patients who started adjuvant therapy within ≤ 12 weeks (blue) or after > 12 weeks (green) following surgery, stratified by clinical stage (I–III). Numbers above the bars indicate the number of cases in each category.

Immediate breast reconstruction was associated with a 45% reduction in the odds of delay (>12 weeks) in the univariate analysis (OR 0.55; 95% CI 0.39–0.76; *p* < 0.01). However, this association was not statistically significant in the multivariate analysis (OR 0.69; 95% CI 0.46–1.01; p = 0.06). Exploratory analyses identified year of surgery and clinical stage as factors associated with delayed treatment in both univariate and multivariate models (**Table 3**). Delays in the initiation of adjuvant therapy were more frequent during the pandemic period (2020 to 2022) compared with the pre-pandemic years (2018 to 2019) (**Figure 2**). The median time to start adjuvant therapy was significantly longer during the pandemic, 13.0 weeks (IQR 10.0–16.0), compared with 11.0 weeks (IQR 8.0–14.0) before the pandemic (p < 0.01). The proportion of patients who began adjuvant therapy more than 12 weeks after surgery was also significantly higher during the pandemic (53.9%) than in the pre-pandemic period (31.4%) (p < 0.01). In addition, clinical stage III emerged as an independent predictor of delayed initiation of adjuvant therapy beyond 12 weeks (**Figure 3**).

**Table 3:** Logistic regression analysis of factors associated with delayed initiation of adjuvant therapy (>12 weeks).

Variable	Category	Univariate		Multivariate	
		OR^a^ (95% CI^b^)	*P*-value	OR (95% CI)	*P*-value
Year of diagnosis					
	2018	Reference		Reference	
	2019	0.59 (0.37 – 0.93)	0.02	0.51 (0.30–0.86)	0.01
	2020	1.88 (1.26 – 2.81)	< 0.01	1.99 (1.27–3.10)	< 0.01
	2021	2.06 (1.39 – 3.04)	< 0.01	2.29 (1.49–3.51)	< 0.01
	2022	2.10 (1.40 – 3.15)	< 0.01	1.96 (1.27 – 3.01)	< 0.01
Clinical stage					
	I	Reference		Reference	
	II	1.74 (0.93 – 3.26)	0.09	1.87 (0.98 – 3.57)	0.06
	III	4.29 (2.29 – 8.03)	<0.01	4.48 (2.33 – 8.60)	< 0.01
Immediate breast reconstruction					
	No	Reference		Reference	
	Yes	0.55 (0.39 – 0.76)	< 0.01	0.69 (0.46 – 1.01)	0.06
Age, years					
	≤40	Reference			
	41to 59	1.03 (0.73 –1.44)	0.87		
	>60	0.90 (0.62–1.32)	0.59		
Ethnicity					
	Black	Reference			
	White	1.10 (0.63–1.94)	0.74		
Comorbidities					
	Absent	Reference			
	Present	1.20 (0.92–1.56)	0.17		
Smoking status					
	Never	Reference			
	Current	1.33 (0.83–2.14)	0.24		
	Former	1.10 (0.72–1.68)	0.66		
Body Mass Index Classification					
	Underweight	Reference			
	Normal	1.25 (0.31–4.95)	0.76		
	Overweight	1.56 (0.39–6.16)	0.53		
	Obesity	1.62 (0.41–6.48)	0.49		
Menopausal status					
	Premenopausal	Reference			
	Postmenopausal	0.96 (0.73–1.26)	0.77		
Hormone receptor					
	Positive	Reference			
	Negative	1.29 (0.97–1.71)	0.08		
HER2^c^ status					
	Positive	Reference			
	Negative	1.00 (0.73–1.37)	0.99		
Histologic grade					
	Grade 1	Reference			
	Grade 2	0.79 (0.50–1.26)	0.33		
	Grade 3	0.85 (0.53–1.38)	0.52		
Distance from city of residence to hospital city, km					
	< 23	Reference			
	23 to 56	1.45 (0.86–2.45)	0.16		
	56 to 361	1.27 (0.92–1.77)	0.15		
	> 361	1.20 (0.87–1.68)	0.27		
Axillary surgery					
	SLNB^d^	Reference			
	ALND^e^	1.11 (0.82–1.49)	0.50		

a OR,odds ratio;

b CI, confidence interval;

c HER2, human epidermal growth factor receptor;

d SLNB, sentinel lymph node biopsy;

e ALND, axillary lymph node dissection; OR, odds ratio. Statistically significant results (p < 0.05) are shown in bold.

#### 3.2.1 Time to adjuvant chemotherapy

Among patients whose first adjuvant therapy was chemotherapy, the mean time to initiation was 10 weeks (± 3.7). Most patients (341, 80.4%) began treatment within the recommended 12-week timeframe. When applying the narrower ESMO-defined interval of 4–6 weeks (6), only 14.6% of patients met this target, with comparable proportions between the IBR and non-IBR groups (18.2% vs. 13.4%, p = 0.28). The median time from surgery to initiation of adjuvant chemotherapy was 9.0 weeks (IQR 7.0–11.0) in the IBR group and 10.0 weeks (IQR 7.0–12.0) in the non-IBR group, with no statistically significant difference between groups (p = 0.08).

#### 3.2.2 Time to adjuvant radiotherapy

For patients who received radiotherapy as the initial adjuvant treatment, the mean time to initiation was 14.3 weeks (± 4.3), with delays occurring in 65.0% of cases (>12 weeks). Patients in the IBR group experienced shorter intervals between surgery and initiation of radiotherapy compared to those in the non-IBR group [13.0 (IQR 11.0–15.0) vs. 14.0 (IQR 11.0–17.0) weeks; p = 0.01].

## 4 Discussion

### 4.1 Impact of delays in adjuvant therapy

Timely initiation of adjuvant therapy remains a critical global benchmark in oncologic care, as treatment delays have been consistently associated with worse clinical outcomes, including increased mortality, recurrence, psychosocial burden, and higher healthcare costs (21). In the present study, 55.7% of patients initiated adjuvant therapy within 12 weeks following surgery, an interval generally accepted by most international guidelines. Hanna et al. (14) demonstrated in a meta-analysis that each four-week delay in initiating surgery, chemotherapy, or radiotherapy was associated with increased mortality risk for several cancer types, including breast cancer.

Several hypotheses have been proposed to explain the oncologic benefits of initiating adjuvant therapy as early as possible after surgery. Prompt treatment may reduce the opportunity for residual cancer cells to proliferate (22, 23), inhibit angiogenesis, prevent the development of drug resistance, limit the acceleration of micrometastatic dissemination following tumor, and mitigate the immunosuppressive effects of the primary tumor (18, 24). This highlights the critical need for timely and coordinated planning between surgery and adjuvant therapy by the multidisciplinary cancer care team. Such synchronization can be especially challenging in low- and middle-income countries, where systemic resource constraints and infrastructure gaps may hinder optimal treatment sequencing.

### 4.2 Immediate breast reconstruction on time to adjuvant therapy

The concern that IBR may delay the initiation of adjuvant therapy stems from its association with higher postoperative complication rates compared to mastectomy alone (5, 12, 13). However, our study found the opposite: among women with stage I–III breast cancer, those who underwent mastectomy with implant-based IBR began adjuvant therapy significantly earlier than those treated with mastectomy without reconstruction.

These results differ from several prior studies that suggested IBR may delay adjuvant therapy (3, 25–29). For instance, Heeg et al. (25) reported that patients who underwent IBR were less likely to initiate chemotherapy within six weeks, although the difference was not significant at nine or twelve weeks. In contrast, other studies have demonstrated comparable or even improved timelines among patients undergoing IBR (4, 20, 24, 30). A systematic review by Harmeling et al. (7) found that IBR does not lead to clinically meaningful delays in chemotherapy. Similarly, Jabo et al. (24) found no significant differences in the time to chemotherapy or radiotherapy among over 56,000 women with stage 0–III breast cancer, including 13,738 who received IBR. The conflicting results across studies may be attributed to the evaluation of heterogeneous patient populations, variation in breast reconstruction techniques, and varied criteria or conceptual frameworks used to define and measure delays in adjuvant therapy initiation.

The discrepancy in findings across studies may reflect patient selection biases. In our cohort, patients who underwent IBR were significantly younger, more often premenopausal, and had fewer comorbidities—factors that may contribute to faster postoperative recovery. This aligns with existing literature indicating that favorable baseline characteristics are common among patients undergoing IBR (4, 24). Despite the higher risk of surgical complications associated with IBR, a meta-analysis by Shen et al. (5) concluded that it does not compromise oncologic safety and may even be associated with improved survival, potentially serving as a proxy for better socioeconomic status.

The reconstructive technique employed may also have influenced our findings. Implant-based breast reconstruction, which was used in all patients in our IBR group, is generally associated with shorter operative times, quicker recovery, and fewer complications than autologous flap procedures (9). Notably, 67% of reconstructions in our study were direct-to-implant, which may have contributed to the shorter interval before adjuvant therapy. In support of this, Kupstas et al. (27) observed greater delays in patients receiving tissue expanders compared to direct-to-implant reconstructions.

When stratified by the type of initial adjuvant therapy, the majority of women (85.4%) commenced chemotherapy within 12 weeks, aligning with the upper limit most frequently endorsed in the literature. There is no international consensus regarding the optimal time interval for initiating chemotherapy or radiotherapy (21). For chemotherapy, the acceptable delay between surgery and treatment generally ranges from 7 to 12 weeks, though this definition remains controversial (26). According to the European Society for Medical Oncology (ESMO), the ideally recommended interval for starting chemotherapy is between 4 and 6 weeks, and evidence suggests that exceeding 12 weeks compromises treatment efficacy (6).

Conversely, while chemotherapy delays were relatively uncommon (14.6%), delays in initiating radiotherapy were observed in 65% of patients, raising concern about procedural bottlenecks specific to radiation planning and access. Determining the optimal interval for initiating radiotherapy following breast surgery remains a subject of debate, as the literature presents heterogeneous findings across different populations and employs varied definitions of what constitutes a delay (31–35). Nevertheless, there is broad agreement that radiotherapy delays can produce adverse outcomes comparable to those associated with chemotherapy delays, even if the specific acceptable timelines remain unclear (36, 37). A meta-analysis found that delays of more than eight weeks were associated with increased locoregional recurrence, although other studies reported no significant detriment for delays extending up to 20 weeks (4). According to the Central and Eastern European consensus, radiotherapy should be initiated after proper wound healing—ideally between 4 and 6 weeks, and no later than 12 weeks postoperatively (19).

Alongside increased rates of postmastectomy breast reconstruction, there has been a parallel rise in the use of postmastectomy radiotherapy (PMRT) among reconstructed patients (38). However, most existing studies have focused on the impact of radiotherapy on breast reconstruction outcomes (39) rather than evaluating how reconstruction may influence the timing of radiotherapy itself. Future research should aim to clarify the optimal timing of PMRT and assess its oncologic consequences more systematically.

### 4.3 Influence of clinical and demographic variables on time to adjuvant therapy initiation

Although IBR was associated with reduced odds of delay in univariate analysis (OR 0.55), this association lost statistical significance after adjustment (OR 0.69, *p* = 0.06), suggesting the presence of confounding variables. Notably, the year of surgery and clinical stage were significant predictors of delay. The COVID-19 pandemic period (2020–2022) emerged as a strong predictor of treatment delay, with 53% of patients experiencing postponed initiation of adjuvant therapy. This observation is consistent with international reports indicating that pandemic-related disruptions significantly compromised access to cancer care. Previous studies have documented interruptions in oncologic treatment delivery influenced by the pandemic’s stage, regional policy responses, and healthcare system capacity (40, 41). Supporting our findings, national data from Brazil reported a 25% reduction in the initiation of adjuvant therapy during this period, particularly among patients with early-stage breast cancer, reinforcing the pandemic’s negative impact on timely treatment (42). Conversely, data from the United States showed no significant delay in systemic treatment initiation (43). Similarly, Losurdo et al. (44) reported that high-volume Italian breast centers participating in the Senonetwork maintained standard treatment timelines without observable delays in surgery, radiotherapy, or systemic therapy. These contrasting findings likely reflect differences in healthcare infrastructure, organizational efficiency, and resource allocation across health systems.

In addition to demographic, socioeconomic, comorbidity-related, and tumor-specific factors, such as histologic subtype, tumor grade, and clinical stage, several variables have been associated with delays in the initiation of adjuvant therapy (45). In our cohort, clinical stage III breast cancer was found to be a significant predictor of delayed treatment. Notably, the non-IBR group exhibited a significantly higher proportion of stage III disease compared to the IBR group (50.3% vs. 24.4%, p < 0.01), which may partially explain the increased incidence of delay in that subgroup. This finding aligns with prior reports suggesting that greater tumor burden may complicate treatment coordination and prolong postoperative recovery. However, contrasting evidence exists. For example, Chavez-MacGregor et al. (18) found that stage III patients experienced fewer delays than those with stage I disease, possibly reflecting a greater sense of clinical urgency that accelerates treatment in some contexts. Despite this, delays beyond 61 days in initiating adjuvant therapy among patients with stage III disease have been linked to worse oncologic outcomes, including higher recurrence rates (46). Additional factors known to influence treatment timelines include patient age, hospital type, communication with physicians, trust in the oncologist, and rural residence (47). These findings underscore the importance of understanding how these intersecting variables contribute to treatment disparities and highlight the need for targeted strategies to promote equitable, timely access to breast cancer care.

Although this study was not designed to assess racial disparities, the cohort’s composition, with 91% of participants identifying as Black women, deserves attention. Nearly half of the patients (44.3%) experienced delays greater than 12 weeks in starting adjuvant therapy, and 45.1% presented with stage III disease, reflecting a high burden of advanced cancer and challenges in timely care among racially minoritized populations. Despite the limited number of White patients precluding direct racial comparisons, our findings align with prior reports showing that Black women face greater barriers to timely breast cancer treatment (48, 49).

In Brazil, structural inequities, racial identity, and socioeconomic status remain persistent obstacles to equitable breast cancer treatment (50). A population-based study found that non-White women, patients with low educational attainment, and those treated within the public health system had significantly longer wait times to initiate treatment (51). Internationally, similar disparities have been well documented. In the United States, Black and Hispanic women, as well as individuals from lower socioeconomic strata, face higher risks of treatment delay, often driven by systemic barriers such as residential segregation and healthcare inaccessibility (52). The U.S. Preventive Services Task Force has reported that, despite comparable screening rates, Black women encounter longer delays in diagnostic follow-up and treatment initiation, which contributes to poorer survival outcomes (53). Furthermore, even within the same tertiary care institutions, racial and ethnic minority patients experience treatment delays, suggesting entrenched institutional biases and structural inequalities (54, 55).

These findings reinforce previous reports and underscore the importance of addressing racial and social inequities in breast cancer treatment. Future investigations should adopt robust methodological designs to explore actionable barriers and facilitators of care. Additionally, implementing patient navigation strategies and equity-centered policies may serve as interventions to ensure more timely and just cancer care delivery across diverse populations.

### 4.4 Time-to-treatment as a quality indicator in breast cancer care

Timely initiation of cancer treatment is widely recognized as a critical factor for both patients and physicians. However, evaluating treatment timeliness is challenging due to the multimodal nature of cancer therapy and variability in treatment pathways, particularly surgical approaches (27). The paradox lies in the fact that prolonged waiting times to initiate treatment negatively impact morbidity, mortality, and patient well-being (56). Accordingly, investigating treatment timing is crucial, as it serves as a performance indicator for healthcare systems and cancer centers. Several organizations advocate for the use of time-to-treatment as a benchmark for breast cancer care quality of care in breast cancer, including the National Quality Measures for Breast Centers Program, the National Accreditation Program for Breast Centers, the National Quality Forum, and the American Society of Breast Surgeons (48).

Despite growing recognition of its importance, the precise impact of treatment delays on breast cancer survival remains insufficiently understood. Understanding the factors that may interfere with treatment logistics—such as the different surgical options for breast cancer—can help inform the design of healthcare systems and care delivery models. Consequently, this may promote equitable and accessible treatment strategies that positively influence oncologic outcomes, cost-effectiveness, and especially, psychosocial well-being.

### 4.5 Strengths and limitations

This study presents several limitations, chiefly its retrospective design, which constrained the analysis to associations between clinical variables and the timing of adjuvant therapy initiation. Consequently, causal inferences could not be established. Data were collected from the electronic medical records of a single tertiary cancer center. The analysis was restricted to the interval between surgery and adjuvant therapy, without evaluating the potential impact of treatment delays on oncologic outcomes.

Additionally, the exclusion of endocrine therapy may limit the applicability of our findings to hormone receptor–positive tumors. As a result, this exclusion may have increased the relative proportion of more aggressive subtypes, such as HER2-positive or triple-negative breast cancers.

Furthermore, another important limitation is that a substantial proportion of patients did not initiate adjuvant therapy within the recommended timeframe. In our cohort, only 55.7% of patients started any adjuvant treatment within 12 weeks after surgery, and among those receiving chemotherapy, only 14.6% initiated treatment within the 6-week interval recommended by ESMO. This deviation from the ideal guideline-based timelines reflects the reality of clinical practice in our setting. Therefore, our findings should be interpreted as representative of real-world conditions.

Nevertheless, this study also exhibits important strengths. The predominance of Black women in the study population is particularly noteworthy, given the persistent underrepresentation of this group in oncologic research. Moreover, the study was conducted at a public cancer referral center with a substantial sample size, enhancing the representativeness and generalizability of the findings. This enabled the evaluation of healthcare quality indicators and the identification of modifiable factors that may inform navigation strategies aimed at reducing treatment inequities, particularly among women with locally advanced breast cancer. Finally, the inclusion of patients who had undergone the same standardized technique for IBR strengthened the internal validity and comparability of the subgroup analyses.

## 5 Conclusion

In this real-world cohort with predominant representation of Black women, patients with clinical stage I to III breast cancer who underwent IBR after mastectomy had a shorter time to the initiation of first adjuvant therapy compared with those who had mastectomy without reconstruction. After adjusting for clinical and demographic confounders, this association was no longer significant, indicating that IBR was not independently associated with delayed initiation of chemotherapy or radiotherapy. These findings suggest that IBR can be safely integrated into treatment planning without compromising timely access to adjuvant care. Nonetheless, the year of surgery and clinical stage III were identified as independent predictors of delayed initiation of adjuvant therapy.

Future research should investigate how demographic, clinical, and pathological factors influence care coordination and treatment logistics throughout the oncologic continuum. Although our study did not directly assess this issue, our findings may nonetheless offer insights to inform the development of navigation programs or targeted interventions aimed at the early identification of women at increased risk for treatment delays. Moreover, it is essential to explore how delays between treatment modalities affect oncologic outcomes and healthcare expenditures, particularly within public health systems.

## Data Availability

The raw data supporting the conclusions of this article will be made available by the authors, without undue reservation.
